# Approaches for implementing society-led community interventions to mitigate snakebite envenoming burden: The SHE-India experience

**DOI:** 10.1371/journal.pntd.0009078

**Published:** 2021-02-25

**Authors:** Priyanka Kadam, Stuart Ainsworth, Freston Marc Sirur, Dhirubhai C. Patel, Jeevan Jonathan Kuruvilla, Dayal Bandhu Majumdar

**Affiliations:** 1 Snakebite Healing and Education Society, Mumbai, India; 2 Centre for Snakebite Research and Interventions, Department of Tropical Disease Biology, Liverpool School of Tropical Medicine, Liverpool, United Kingdom; 3 Department of Emergency Medicine, Kasturba Medical College, Manipal Academy of Higher Education, Manipal, India; 4 Shree Sainath Hospital, Dharampur, India; 5 Gandhiji Health and Development Centre, Barwadih, Jharkhand, India; 6 Calcutta National Medical College Hospital, Kolkatta, India; Universidad de Costa Rica, COSTA RICA

The burden due to snakebite envenoming in India is unparalleled, with deaths estimated to be in the region of 58,000 per year and scores more victims left permanently disabled or disfigured [1,2]. The demographics of typical victims in most Indian states burdened with snakebite incidences are ones of poverty, illiteracy, and preexisting poor health and comorbidity [3,4].

The recent World Health Organisation snakebite envenoming strategy defines 4 key objectives in order to achieve the goal of reducing snakebite death and disability by 50% by 2030 [5,6]. One of the identified objectives for attaining this ambitious target is “empowering and engaging local communities,” based on strong evidence that disease interventions are more likely to succeed through active engagement with local communities [7]. It is this objective where civil society initiatives can make substantial impact.

One such civil society initiative is the Snakebite Healing & Education Society of India [SHE-India, (www.she-india.org)]. Initially started as a personal project to provide a voice for India’s snakebite victims, SHE-India has grown into a nationwide, multipronged grassroots organisation which campaigns for and delivers effective healthcare and education and to reduce human-snake conflicts. To facilitate this, SHE-India engages and works with all levels of Government and non-Government partners from national to local level (Fig 1). As of April 2020, SHE-India is actively working to reduce snakebite burden within the states of Maharashtra, Jharkhand, West Bengal, Karnataka, Uttar Pradesh, Gujarat, Chhattisgarh, and Rajasthan.

**Fig 1:**
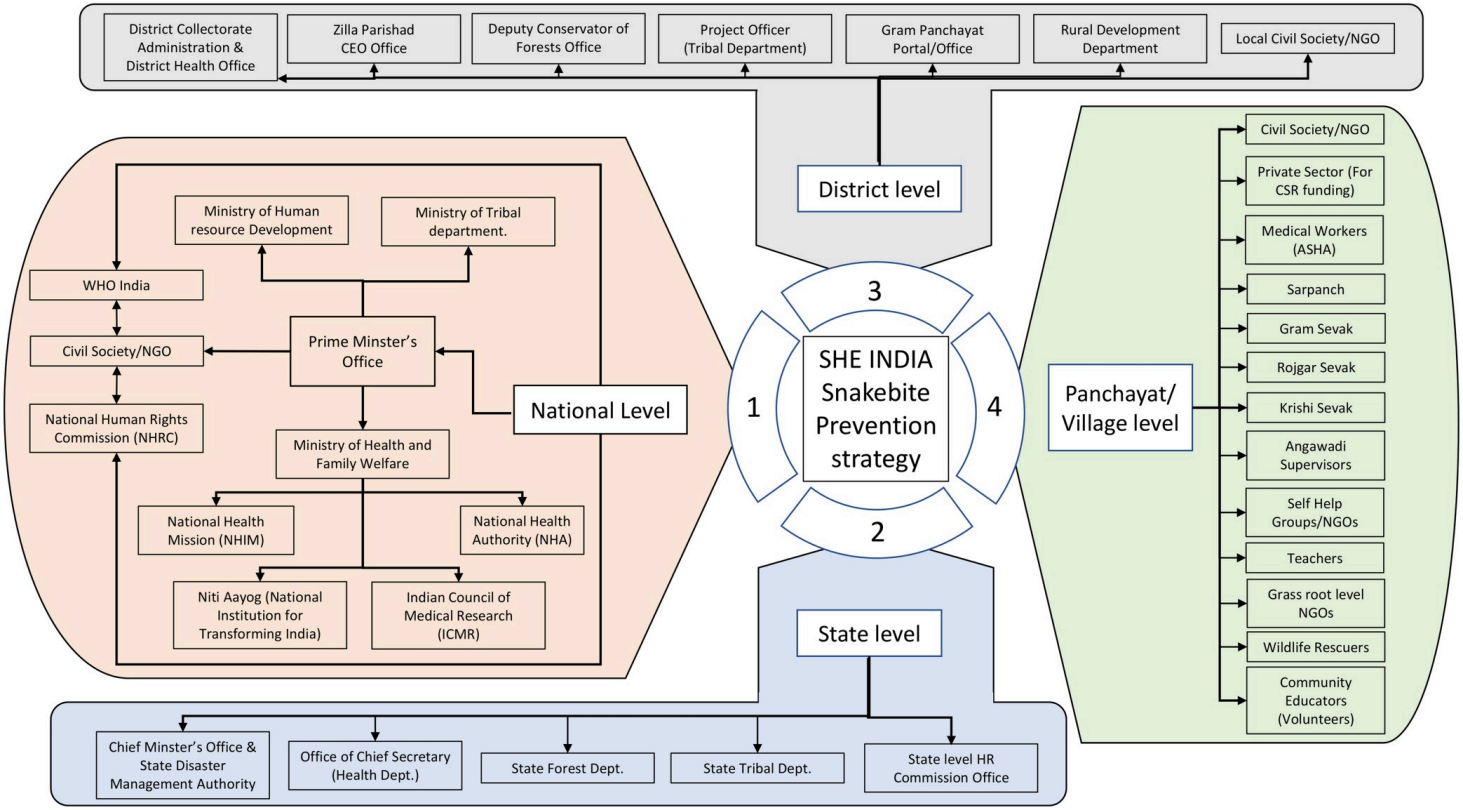
Flow chart outlining SHE-India’s working model for engagement of key stakeholders at different levels. Zilla Parishad = District level council. Sarpanch = Village head. Gram Sevak = community welfare advisor. Gram Panchayat = village/town council. Rojgar Sevak = Village council employees. Krishi Sevak = Agricultural advisor. Angawadi supervisors = Supervisor of Preschool (<5 y) educators.

SHE-India actively recruits and coordinates a group of expert volunteer collaborators representing a diverse range of expertise, including clinicians, lawyers, ex-bureaucrats, herpetologists, scientists, teachers, snake rescuers, government officials, and other professionals. Thus, SHE-India provides a platform where anyone interested in tackling snakebite, can contribute in some way, allowing pooling of resources and expertise for a more concerted and effective response. All volunteers undergo training and use educational materials produced by SHE-India to ensure the basic message of snakebite prevention and first aid is consistent, accurate, and safe.

Despite the rapid growth and successes of SHE-India, it receives and operates on meagre funding. This limits the scale of activities possible—a situation no doubt common in resource poor settings across the tropics. Accordingly, SHE-India has developed interventions that are affordable, accessible, and impactful. Here we detail SHE-India’s experiences with low-resource, community-based actions which have been implemented to confront India’s snakebite challenge. These initiatives can be applied to many low- and middle-income countries with endemic snakebite problems.

## The role of social media

The near-ubiquitous use of mobile phones providing internet access has transformed the way the world communicates. Mobile internet use in 2016 accounted for 66% to 82% of internet traffic in India, Indonesia, South Africa, and Nigeria [8]. Social media applications provide powerful advocacy and educational platforms as they are free, easy to use, and permit enormous reach to audience of potentially billions. Therefore, any local, low-cost community intervention for Snakebite envenoming will undoubtedly make use of some form of social media. SHE-India is heavily active across social media platforms such as Facebook (@SnakebiteHealing), Twitter (@SHEINDIA1), and YouTube (youtube.com/SnakebiteHealingEducationSociety), where it shares awareness and training in the form of educational videos and posters on snakebite prevention, correct first aid, and news of the snakebite scenario in India.

Many of the interventions described below strongly rely on the use of social media for distribution of materials and engagement with at-risk populations.

It is important to consider that the sharing of any material which may identify individuals is likely to be governed by local laws. SHE-India obtains consent before using any personally identifiable information relating to victims of snakebites prior to publication.

## Education for snakebite prevention and first aid

A key activity SHE-India has undertaken is to create a short (5 min), clear educational video on snakebite prevention, first aid, safe transport to hospital, and interventions to avoid, such as the use of tourniquet, cutting and attending traditional healers. Crucial for wide dissemination, this video is narrated in 12 regional languages of India allowing increased audience reach (28.7K views as of December 2020 on YouTube). The videos are freely available on multiple social media platforms and are further disseminated via snake rescuer groups through social media channels, Forest Department awareness programs, educational institutions, community groups, and local and district government offices and departments. Links to download the videos have been shared with more than 200 volunteer groups from across India, which have further used the snakebite prevention video in their own community programs.

**Fig 2:**
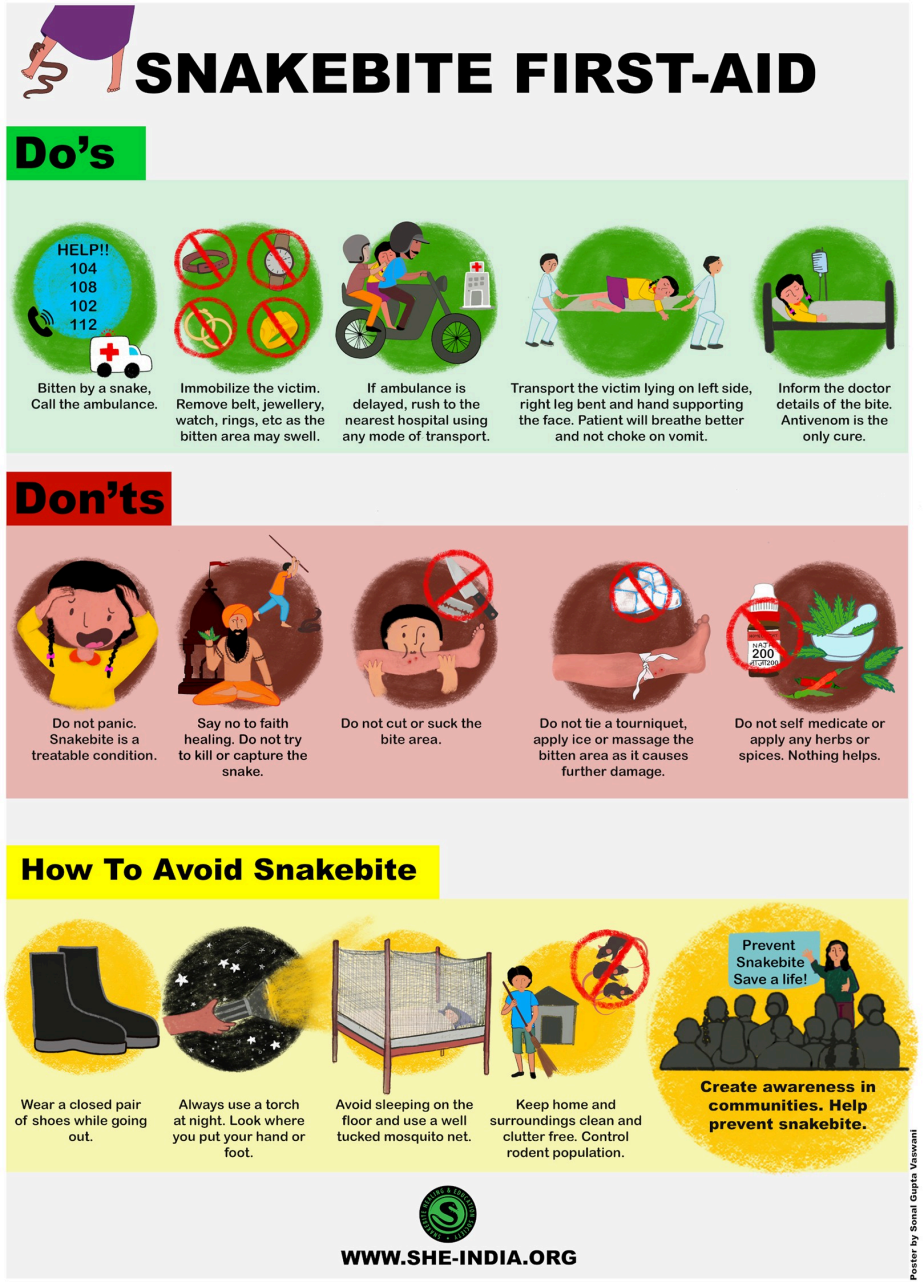
Example of clear, illustrative poster on snakebite awareness. This poster is available in 6 regional languages of India and aims to translate into further 11 languages.

**Fig 3:**
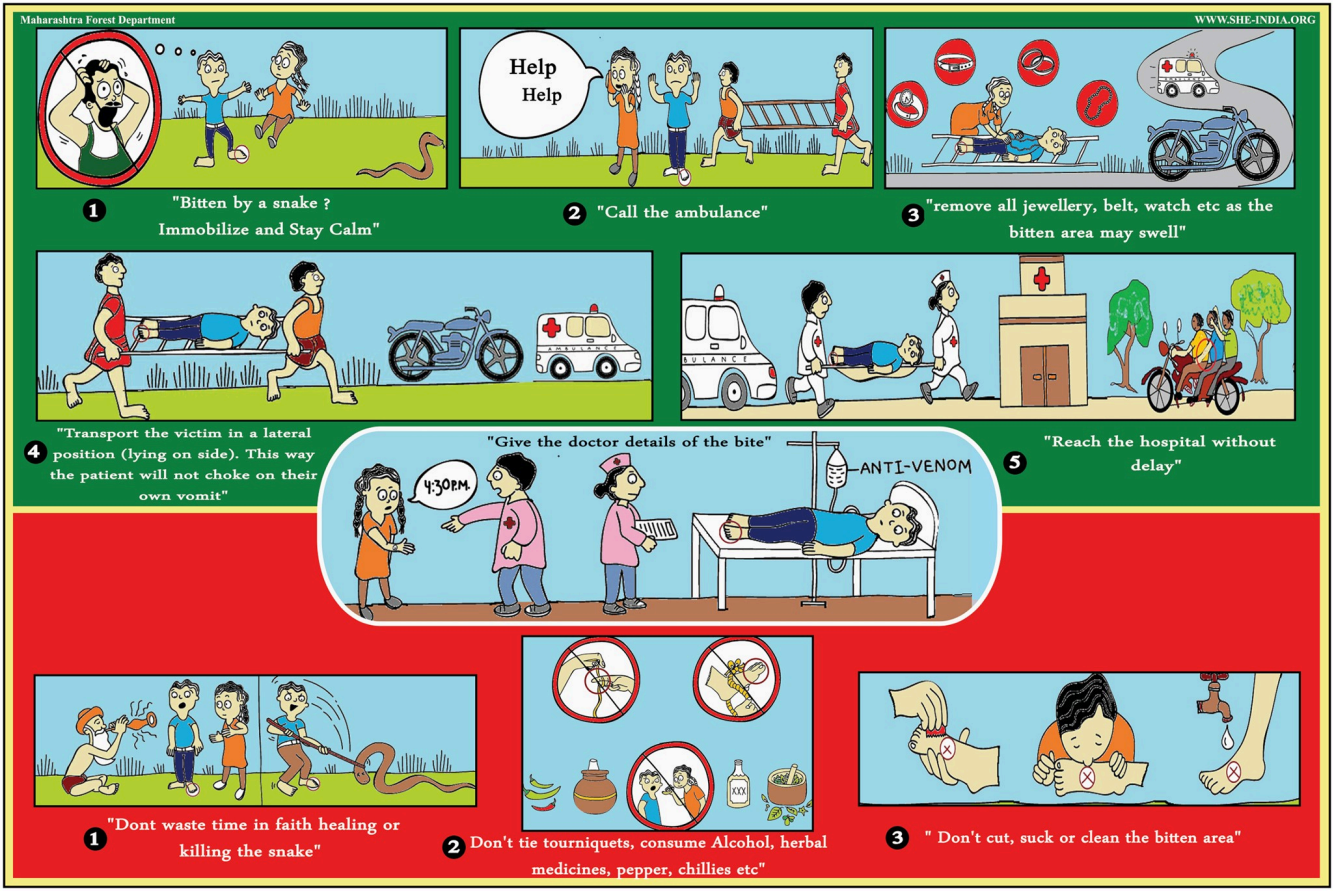
Example of clear, illustrative poster on snakebite awareness. This poster is available in 12 regional languages of India.

To compliment these videos, SHE-India has also created illustrative posters regarding snakebite first aid “Do’s and Don’ts” (Figs 2 and 3) available in 12 regional languages. The use of clear illustrative posters is essential in distributing such information to the impoverished and frequently illiterate rural communities. Posters are distributed and displayed in strategic, high-footfall places by local volunteers. Additionally, all material is freely distributed on several social medial platforms and by other stakeholders, further increasing reach. It is critically important that such videos and posters contain factually correct information and best practices and thus any such material must be produced in partnership with experienced snakebite clinicians and local herpetologists.

## Engagement to empower and improve local healthcare

It is extremely important that if ground-level interventions are to have an impact, they are communicated by people or groups whom rural communities are familiar with and trust. Village-level stakeholders, such as the village headman, and community advisors (Fig 1), are actively engaged by SHE-India for promoting awareness and prevention of snakebites. Furthermore, SHE-India provides snakebite-specific healthcare training and awareness materials to the women-led accredited social health activist (ASHA) workforce. ASHA is group of rural healthcare professionals which are known by every village family and are trusted intimately, thus allowing effective dissemination of snakebite awareness in villages across rural India.

SHE-India routinely educates village volunteers on snakebite prevention and first aid training, creating local first responders to assist families in seeking essential immediate medical treatment in case of a snakebite. In the last 5 years, approximately 1,200 such volunteers have been trained via live workshops and virtual seminars. Similar training has been provided to an additional approximately 2,250 forest department staff and volunteers in the state of Maharashtra and Gujarat.

## Traditional/faith healers

Attempting to collaborate with local traditional healers is important as they command respect in the communities and anything that they propagate will naturally be well received. Indeed, engaging with traditional healers has proved successful in some countries for some diseases [9]. SHE-India has attempted to engage with local traditional healers in the state of Rajasthan and Jharkhand in order to improve snakebite awareness and ultimately reduce the time taken for snakebite victims to present to health facilities. Unfortunately, such efforts have so far been unsuccessful. For example, in 2015, SHE-India attempted to bring together faith healers in Sawai Madhopur, Rajasthan, with the assistance of the local authorities as an intermediary, to attempt discussion on snakebite treatment. Despite many attempts and support from the local government officials, traditional healers shied away from attending the workshop.

This experience highlights that engaging traditional healers can be extremely difficult and time consuming, and while clearly worthwhile in some regions, it may be more productive to focus on other community interventions, such as first aid and health education around misconceptions in traditional healing for snakebite, thus allowing the patients an informed choice [10]. This appears to be anecdotally the case in SHE-India’s experience, as some communities we have engaged with appear to have become more proactive in taking snakebite patients directly to the hospital instead of first visiting a traditional healer.

## Advocacy

The wealth of a country is its citizens. The segment of society that produces the country’s food, runs its industries, and builds key infrastructure is unfortunately the most exposed to snakebites [11]. It is this population of agricultural workers and labourers who are directly connected to a country’s GDP. It has been demonstrated that snakebite prevention and effective treatment is highly economically productive [12,13]. The issue should be therefore discussed at the highest level of the country’s leadership.

The successes of the simple, low-cost interventions outlined here and employed by SHE-India have provided an authoritative voice for the most marginalised Indian snakebite victims. Production of an award-winning, hard-hitting, freely available low-budgeted documentary film, titled *The Dead Don’t Talk* [14], which simply records the stories and reality of the misery that snakebite in India inflicts, has proved a particularly useful advocacy tool, motivating many more people with influence to work on mitigation plans to reduce snakebite burden in India.

SHE-India has used this hard-earned influence to engage with the Health Ministry at central and state levels to advocate and recommend policy actions to mitigate snakebites and leverage the existing health schemes. However, until governments ultimately take responsibility and ownership of these issues, it will remain up to local nongovernment organisations, civil societies, and academics to try and stem the misery.

The impact of low-cost interventions can be measured only by a sustained funded study over an extended period. An important next step is to assess the impact of society-led community interventions to mitigate snakebite envenoming burden with a mixed method approach. We are actively pursuing such studies.

## Conclusion

The simplest methods to reduce snakebite burden is through community engagement—prevention is easier than cure. The strategic approaches outlined here, while basic in their application, are anecdotally effective and low resource and can be applied strategically across various states in India and potentially in other countries suffering substantial snakebite incidence. Use of these low-resource approaches has thus far reached millions of people across India.

While anecdotally the impact is clear, a single life, limb, or livelihood saved would represent a success for any civil society initiative, the assessment of the larger scale impact of such efforts is challenging. Only a sustained funded study over an extended period can determine the effect of the work done and identify long-term and enduring positive outcomes resulting from awareness programs.

SHE-India’s experience demonstrates that civil society initiatives can make positive impacts in preventing snakebite and improving victim outcomes with little resource.
